# Phenotypic and Molecular Characterization of Carbapenems Resistant *Escherichia coli* Isolated from Patients with Urinary Tract Infections in Ardabil Province, Iran

**DOI:** 10.30699/IJP.2022.538613.2716

**Published:** 2022-08-11

**Authors:** Shabnam khavandi, Mohsen Arzanlou, Roghayeh Teimourpour, Hadi Peeridogaheh

**Affiliations:** Department of Microbiology, School of Medicine, Ardabil University of Medical Sciences, Ardabil Iran

**Keywords:** Carbapenemase, Escherichia coli, Urinary tract infections

## Abstract

**Background & Objective:**

Carbapenem-resistant is Gram-negative bacteria representing a worldwide public health problem. The present study aims to survey the phenotypic and genotypic characteristics of carbapenem-resistant *Escherichia coli* isolates collected from hospitalized patients and outpatients in Ardabil province, Iran.

**Methods:**

Two hundred samples were collected from the patients who had already been referred to the hospitals in Ardabil, Iran, from January to June 2017. Each patient's social and demographic data were recorded in the first step. The resistance profile of all* E. coli* isolates against imipenem and meropenem antibiotics were determined using the Kirby-Bauer disk diffusion method. Moreover, the broth microdilution method determined the Minimum Inhibitory Concentration (MIC) of E. coli isolates to imipenem. The Carbapenem Inactivation Method (CIM) and Carba NP test were employed for screening carbapenem-resistant strains. The frequency of carbapenem-encoding genes was determined using Polymerase Chain Reaction (PCR) method. The Enterobacterial Repetitive Intergenic Consensus (ERIC)-PCR analysis was used to evaluate the genetic relatedness of *E. coli* isolates.

**Results:**

Out of 200 urine samples, 66% (n = 132) of the samples were collected from women. The patients' age varied from 1 month to 93 years. Results of the disk diffusion method revealed that 33% (n=66/200) of *E. coli* isolates were resistant to imipenem. However, imipenem resistance was detected in 37% (n = 74/200) of the *E. coli* isolates using broth microdilution method. All *E. coli* isolates were negative in CIM and Carba NP tests. Moreover, we could not detect any carbapenemase encoding genes among *E. coli* isolates. The ERIC-PCR method revealed the *E. coli* strains were classified into 39 clusters with 80% similarity.

**Conclusion:**

It appears that *E. coli* is the most common cause of urinary tract infection in Ardabil province.

## Introduction

The emergence and spread of Carbapenem-resistant *Enterobacteriaceae* (CRE) constitutes a major threat to public health (1). Carbapenem antibiotics contain a β-lactam ring with a combined hydroxyl-substituted chain that plays an essential role in controlling infections caused by Gram-Negative Bacteria (GNB) (2). Carbapenems generally have the broadest spectrum of activities and the highest potency against all GNB and Gram-positive bacteria (2). These antibiotics are the last resort for patients who are severely ill or suspected of being infected with ESBL and AmpC-resistant bacteria (3) In recent years, resistance to carbapenems has been increasingly reported in different countries (4, 5). The main resistance mechanisms against carbapenems are as follows: 1) reduction of permeability; 2) efflux pumps; 3) changes in Penicillin-Binding Protein (PBP); and 4) ß-lactamases production (6). The organisms that hydrolyze carbapenems frequently resist other antibiotics, such as aminoglycosides, fluoroquinolones, and sulfonamides. Therefore, treating these infections is very difficult due to the increasing resistance to Carbapenem antibiotics (3). 

In 2013, the Centers for Disease Control and Prevention (CDC) categorized CRE as one of the three urgent antimicrobial resistance threats. Moreover, in 2017, the World Health Organization (WHO) identified CRE on the priority pathogen list for further research and discovery of new antibiotics (7). Globally, bacteria belonging to the *Enterobacteriaceae* family are the main agents of nosocomial and community-acquired Urinary Tract Infection (UTI) (8). UTI is among the most prevalent infectious diseases, along with upper respiratory tract infection (8). It is often associated with a high mortality rate; annually, 150 million people are identified with UTI statements (9). In* the Enterobacteriaceae* family, the *Escherichia coli* and the *Klebsiella* *pneumoniae* are the most common causes of UTI (8).

Regarding the acceleration of the horizontal transferring of resistance genes (10), Carbapenemase-related resistance genes have spread among bacteria due to non-standard prescriptions and excessive usage of antibiotics, particularly in developing countries (11).

Considering the role of carbapenem-resistant organisms in causing infection in the hospital and community, assessing the resistance pattern of carbapenem-resistant strains using the standard laboratory methods as well as developing a comprehensive program for management and control of antibiotic use are urgently needed. Therefore, the current study aimed to survey the phenotypic and genotypic characteristics of carbapenem-resistant *E. coli* isolates collected from hospitalized patients and outpatients in Ardabil province, Iran.

## Material and Methods

**Sampling and Isolation of** ***E. coli***

Two hundred urine samples were collected from patients who had already been referred to hospitals affiliated with Ardabil University of Medical Science, Ardabil, Iran from January to June 2017. In the first step, the written consent form was obtained after explaining the purpose of the study to patients or nurses. A questionnaire was designed for each patient, and social and demographic data, including gender, age, residence (urban or rural), and travel history, were collected. In the next step, morning urine samples were cultured first on blood agar and MacConkey agar medium (Merck, Germany). All plates were incubated for 24 hours at 37°C. After 24 to 48 hours, microbial growth was observed and documented. Urine culture was granted positive with >10^5^ cfu/mL. 

The detection and identification of *E. coli* isolates were performed using the gram staining method and conventional biochemical tests, including oxidase and catalase tests, growth on Triple Sugar Iron (TSI) Agar, H2S and gas production, SIM (Sulphide Indole Motility) test, IMVIC test (Indole, Methyl red, Voges Proskauer, and Citrate), motility, and urea tests.

DNA extraction was performed by using the boiling method according to the study performed by Azimi* et al.* (12). The final identification of *E. coli* isolates was carried out by Polymerase Chain Reaction (PCR) method using specific primer pair targeting 16s rRNA gene.

**Antimicrobial Susceptibility Testing**

The resistance profile of all* E. coli* isolates against imipenem and meropenem antibiotics (MAST Company) was determined using the Kirby-Bauer disk diffusion method. Moreover, the Minimum Inhibitory Concentration (MIC) of *E. coli* isolates against imipenem (Sigma (Sigma–Aldrich, cat No. PZ0021) was determined using the broth microdilution method. The results of the antibiotic susceptibility test were interpreted according to the Clinical and Laboratory Standards Institute (CLSI) criteria (13) and determined as either resistant (R), intermediate (I), or susceptible (S).

**Carbapenem Inactivation Method (CIM)**

Based on the CLSI guideline (13, 14), the Carbapenem Inactivation Method (CIM) was used to trace the activities of Carbapenemases in *E. coli* isolates. Based on the CIM test, a full 10 μL loop of pure culture was taken from a Mueller-Hinton agar plate (Merck, Germany), and the pure suspension was prepared in 400 μL water. At the next step, a 10 μg meropenem disk (MAST Company) was immersed in the suspension of the carbapenemase enzymes produced from bacteria and incubated for 18-24 hours at 35°C + 2°C. Following overnight incubation, the meropenem disk was removed from the suspension. Mueller-Hinton agar plates were inoculated with a carbapenem-susceptible *E. coli* strain (ATCC 29522), and meropenem disks were placed on these plates. Plates were incubated at 35°C for 18 to 24 hours. The inhibition zone >19 mm was considered a negative result.

**Carba NP Test**

The Carba NP test was used for detecting carbapenem-resistant *E. coli* isolates according to standard protocol (CNPt-CLSI). For this purpose, all *E. coli* isolates were cultured on Mueller-Hinton agar and incubated overnight at 37°C. One loop of pure *E. coli* colonies was scraped and suspended in a 1.5-ml Eppendorf tube containing 100 µL of 20mM Tris-HCL lysis buffer and mixed using a vortex device. At the next step, 0.05% phenol red with 0.1 mmol/liter ZnSO4 (pH 7.8 and 6 mg/ml imipenem; Sigma) was used as an aqueous indicator solution and mixed with prepared bacteria lysate (reaction tube). On the other hand, the phenol red solution without antibiotics was used as a control tube. Finally, all tubes were incubated at 35°C for 2 hours, and after that, the color change from red to orange/yellow in the reaction tube was monitored, and results were documented. *K. pneumoniae* ATCC BAA 1705 and *K. pneumoniae* ATCC BAA 1706 were used as the positive and negative control, respectively.

**Detection of Carbapenemase Encoding Genes**

DNA extraction of *E. coli* isolates was performed using a DNA extraction kit [AllPrep DNA minikit (Qiagen, Inc.)] according to the manufacturer's instruction. The quality of the extracted DNA was evaluated using The NanoDrop Spectrophotometer (Thermo, USA). DNA samples with an OD260/OD280 ratio ≥1.8 were applied for further analysis. The presence of the main carbapenemase encoding genes including *bla*_KPC_, *bla*_IMP_, *bla*_NDM_, and *bla*_OXA-48 _was determined by PCR using specific primers. The sequence of the primers with their annealing temperature is listed in Table 1. 

The PCR reaction mixture (25 μL) contained 1 U Taq DNA polymerase (Sinaclone, Iran), 10 ng μL^−1^ of each primer (Sinaclone, Iran), 0.5 mM of each dNTP, 1× reaction buffer, and 1.5 mM MgCl_2_ (Sinaclone, Iran). Amplification was performed in a thermocycler (Biorad, USA) as follows: an initial denaturation (94°C, 5 minutes) followed by 35 cycles of denaturation (95°C, 30 s), annealing (30 s at 55-60°C), and extension (72°C, 30 s) and final extension (72°C, 5 minutes). The PCR products were screened on 1.5% agarose gel (Sigma, USA) stained with ethidium bromide and visualized by a gel documentation system**.**

**Table 1:** PCR primers used for final identification of *E. coli* isolates and amplification of carbapenemase encoding genes

Genes	Primers	Sequence (5′ → 3′)	Annealing temperature	Reference
16S rRNA	F	AGAGTTTGATCMTGGCTCAG	55	**(** 15 **)**
	R	CCGTCAATTCATTTGAGTTT		
*bla*_KPC_	F	TGTCACTGTATCGCCGTC	60	**(** 16 **)**
	R	CTCAGTGCTCTACAGAAAACC		
*bla*_IMP_	F	GAAGGCGTTTATGTTCATAC	58	**(** 17 **)**
	R	GTACGTTTCAAGAGTGATGC		
*bla*_NDM_	F	GCAGCTTGTCGGCCATGCGGGC	59	**(** 18 **)**
	R	GGTCGCGAAGCTGAGCACCGCAT		
*bla*_OXA-48_	F	GCGTGGTTAAGGATGAACAC	56	**(** 19 **)**
	R	CATCAAGTTCAACCCAACCG		

**Enterobacterial Repetitive Intergenic Consensus PCR**

The Enterobacterial Repetitive Intergenic Consensus (ERIC)-PCR analysis was used for evaluation of the genetic relatedness of *E. coli* isolates. Table 2 shows the specific primer pair used in the ERIC-PCR method. The PCR master mix (25 μL) contained 1 μL of bacterial DNA, 10×buffer 2.5 μL, dNTPs (2.5 mmol/L) 0.5 μL, Taq DNA polymerase (5 U/ μL) 0.2 μL, MgCl_2_ (25 mmol/L) 1.5 μL, primers (100 mol/L) each with 1 μL, and distilled water 17.3 μL. PCR reaction was set as in the following condition: initial denaturation of 94°C for 1 min and 29 cycles of 94°C for 30 s, 52°C for 1 min, and 72°C for 4 minutes, followed by a post-extension of 72°C for 5 minutes. Finally, 1.5% agarose gel was used for electrophoresis of PCR products by DNA with a 50bp DNA ladder as a marker.

For constructing a computerized dendrogram, the pattern of bands in gel electrophoresis photos was analyzed by GelCompar II software. The existence of 80% similarity was considered for plotting phylogenetic trees and determining clonal relationships among strains.

**Table 2:** The sequence of the primer used for the ERIC-PCR method

Gene	Primer	Sequence (5′ → 3′)	PCR product size
ERIC-PCR	F	ATGTAAGCTCCTGGGGATTCAC	**Variable**
	R	AAGTAAGTGACTGGGGTGAGCG	

## Results

**Sampling and Patients' Characteristics**

In the current study, 200 urine samples were collected from patients who had already been referred to the hospitals affiliated with Ardabil University of Medical Sciences for 6 months. Results showed that all urine samples were positive for *E. coli* isolates. Out of 200 *E. coli* isolates, 66% (n=132) and 34% (n=68/200) were derived from women and men, respectively. The patients' age varied from 1 month to 93 years. Our analyses revealed that 59% (n=117/200) and 41% (82/200) of *E. coli* isolates were isolated from hospitalized patients and outpatients, respectively. In total, out of the 200 patients included in the present study, 41 (20.5%) patients had a hospitalization history, 93 (46.5%) patients had a history of antibiotic use during six months before admission, and 49 (24.5%) patients had a specific infectious disease.

**Antibiotic Resistance Pattern Analysis**

The results obtained from the Kirby-Bauer disk diffusion method revealed that 33% (n=66/200) and 3% (n=6/200) of *E. coli* isolates were resistant and intermediate to both imipenem and meropenem antibiotics, respectively. Moreover, results showed that 100% (n=200/200) of *E. coli* isolates were susceptible to meropenem antibiotics. In contrast, 64% (n = 128/200) of *E. coli* isolates were resistant to imipenem.

The measurement of MIC of imipenem was performed in the concentrations of 0/125 (μg/mL) to >128 (μg/mL). The MICs of imipenem for *E. coli *isolates are shown in Table 3. Imipenem resistance was detected in 37% (n=74/200) of the *E. coli* isolates using the broth microdilution method.

**Table 3:** Results of minimum imipenem inhibitory concentration (μg/mL)

(μg/mL)	0/125	0/25	0/5	1	2	4	8	16	32	64	128	>128	Total
N	74	12	17	11	12	3	2	4	9	5	15	36	**200**
%	37	6	8/5	5/5	6	1/5	1	2	4/5	2/5	7/5	18	**100**

**CIM and Carba NP test**

**Fig. 1:**
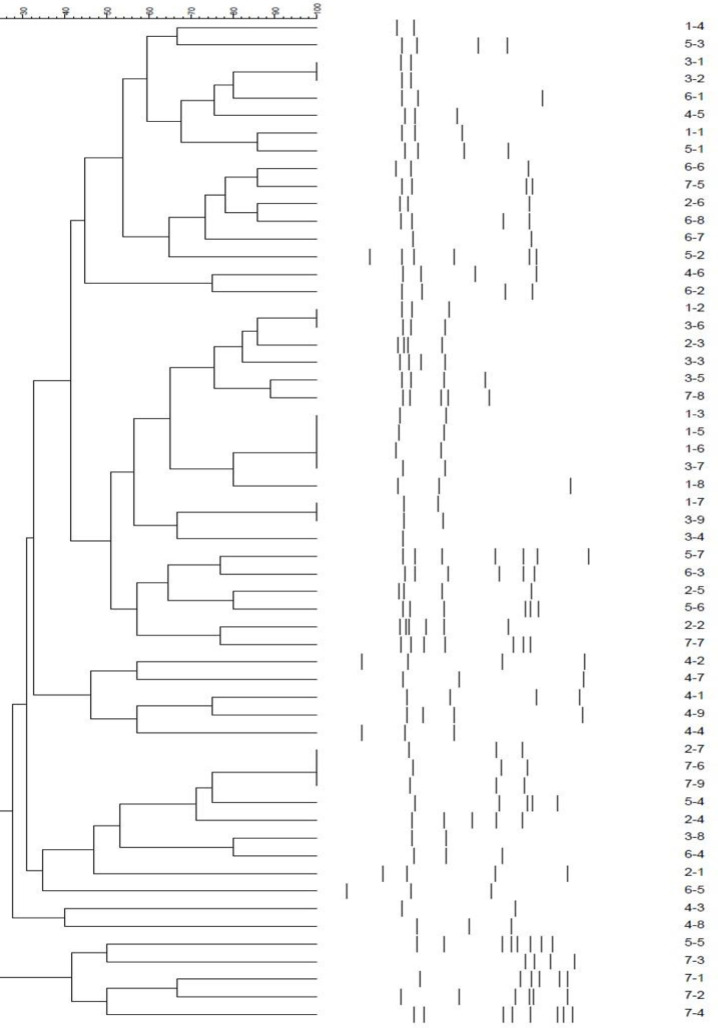
Dendrogram showing ERIC-PCR profiles of all *E. coli* isolates

Results of the CIM test revealed that for all *E. coli* isolates, the inhibition zone of the meropenem disc was more than 19 mm. Therefore, all *E. coli* isolates were negative in the CIM test. Moreover, all *E. coli* isolates were negative in the Carba NP test.

**Frequency of Carbapenemase Genes**

PCR method was applied to detect the carbapenemase encoding genes. We did not detect any surveyed carbapenemase encoding genes, including KPC, IMP, NDM, and OXA-48. Therefore, all E. coli isolates were negative carbapenemase encoding genes.

**ERIC-PCR Analyses**

The ERIC-PCR banding pattern indicated 2 to 8 bands encompassing about 150 bp to 2900 bp. The predominant fragments in DNA fingerprints were determined with a size of bp, and the related dendrogram is shown in Figure 1. The ERIC-PCR detected 80% similarity, and all *E. coli* isolates were classified in 39 clusters.

## Discussion

According to the World Health Organization (WHO) reports, antibiotic-resistant bacteria represent a serious public health threat worldwide. Antibiotic-resistant bacteria account for a high annual hospital death rate (1). Among antibiotic-resistant bacteria, the *Enterobacteriaceae* family frequently appears in healthcare settings and accounts for the high percentage of community- and hospital-acquired infections (20). The bacteria belong to the *Enterobacteriaceae* family that is easily transmitted to humans via different ways (21). *E. coli* is an important member of the *Enterobacteriaceae* family that causes various infections such as UTI, septicemia, pneumonia, peritonitis, and meningitis (22). *E. coli* accounts for about 80% of all UTIs worldwide (22).

In the present study, an attempt was made to survey the phenotypic and genotypic characteristics of carbapenem-resistant *E. coli* isolates collected from hospitalized patients and outpatients in Ardabil province, Iran. Results of our research revealed that out of 200 *E. coli* isolates, 34% and 37% were resistant to carbapenem antibiotics using disk diffusion and broth microdilution method, respectively. However, our findings showed that all *E. coli* isolates were negative in CIM Carba NP tests.

Several studies have surveyed the frequency of phenotypical resistance to carbapenem antibiotics worldwide. In a study by Ripabelli* et al.* in 2018, they found that 70% of the *E. coli* isolated from UTIs were carbapenem-resistant (23). Murugan* et al.* in 2019 in India revealed that the frequency of carbapenem-resistant *E. coli* isolates was 29.03% (24). In contrast, Sahu* et al.* in India reported that all *E. coli* isolated from urine samples were susceptible to imipenem (13). Mahmoud* et al.* from Sudan revealed that 33% of *E. coli* isolates were resistant to imipenem (25). Results of a study performed by Murugan* et al.* showed that 29% of E. coli isolates were resistant to at least one of the surveyed carbapenem antibiotics. They reported that the resistance rate to surveyed antibiotics, including meropenem, imipenem, and ertapenem, was 23.3%, 2.1%, and 1.4%, respectively (24). In 2020, Gurung* et al.* revealed that *E. coli* isolates constituted 28.6% of carbapenem-resistant GNBs (26). Azimi* et al.* from Iran reported that the Carba-NP test identified 11% carbapenem-resistant strains among GNBs (27).

Variation in the frequency of antibiotic resistance and carbapenem-resistant isolates may result from several factors such as sample size, type of samples, the difference in diagnosis method, the difference in materials used, and differences in the type of the infection of patients (28, 29).

Based on the genotypic tests performed by specific primers and PCR method, all *E. coli* isolates were negative for surveyed genes encoding carbapenemase (*bla*_KPC_, *bla*_IMP_, *bla*_NDM_, and *bla*_OXA-48_). Gurung* et al.* revealed that 33.3% of GNBs positive for the *bla*_OXA-48_ gene were *E. coli *(26).

According to Dagher* et al.* from Lebanon, results showed that among *E. coli* isolates, the predominant carbapenemase gene was detected as *bla*_OXA-48 _(30). Based on a study performed by Armin* et al.*, *bla*_OXA-48 _and *bla*_NDM-1 _were the most frequently detected Carbapenemases in GNB (31). The findings of Mahmoud* et al.* agreed with our study's findings. They demonstrated that all *E. coli* isolates were negative for *bla*_NDM_, *bla*_VIM_, and *bla*_IMP_ genes. However, Mahmoud* et al.* demonstrated that bla_OXA-48_ and *bla*_KPC_ genes were detected in 15.5% and 8.8% of the isolates, respectively (25). Ssekatawa* et al.* from Uganda illustrated that the prevalence of *bla*_OXA-48_, *bla*_VIM_, *bla*_IMP_, *bla*_KPC_, *bla*_NDM_ genes was 33%, 21%, 16.5%, 14.8%, and 14.8%, respectively (32).

Given that 37% of the samples were resistant to carbapenem according to MIC results, it can be concluded that other resistance mechanisms may be responsible for carbapenem resistance in this study. The ERIC-PCR patterns were analyzed by GelCompar II software, and interpretation of the results demonstrated that the strains with 80% similarity were classified into 39 clusters. In total, it can be concluded that the ERIC-PCR method for typing was relatively easy, cost-effective, and reliable. 

## Conclusion

The results of our study showed that imipenem resistance was significantly higher in the study area. Phenotypic tests described here are robust and reliable methods for detecting carbapenem-resistant *E. coli *isolates. Because of the absence of carbapenemase genes, it can be represented that resistance results from mutations in the target protein, and the drug's penetration into the organism is reduced or its output increased. According to the rapid growth of antibiotic resistance due to the overuse and non-standard prescriptions of antibiotics, especially in developing countries, evaluation of the resistance pattern of pathogenic organisms and development of a comprehensive program of management and control of antibiotic use are usually necessary. Moreover, it can be said that the ERIC-PCR method is suitable for molecular typing in epidemiological studies, which can be used to determine infection control strategies.

## Ethics Approval

The research was approved by the Research and Ethics Committee of the Ardabil University of Medical Science (Number: IR- 09409585).

## Conflict of Interest

The authors declared no conflict of interest.

## Funding

None.
